# Modified Jiaoqi powder ameliorates ulcerative colitis through gut microbiota-tryptophan metabolism-AhR signaling modulating-ILC2/ILC3 balance

**DOI:** 10.3389/fmicb.2026.1764082

**Published:** 2026-02-25

**Authors:** Ting Jin, Qi Yi, Yingqi Wang, Weiping Liu, Li Zheng, Jinqi An, Hong Mi, Lian Zhou, Fengbin Liu, Xiaojing Wang

**Affiliations:** 1 The First Affiliated Hospital of Guangzhou University of Chinese Medicine, Guangzhou, China; 2 Guangdong Clinical Research Academy of Chinese Medicine, Guangzhou, China; 3 School of Pharmaceutical Sciences, Guangzhou University of Chinese Medicine, Guangzhou, China

**Keywords:** aryl hydrocarbon receptor, innate lymphoid cells, intestinal microbiota, MJQP, ulcerative colitis

## Abstract

**Background:**

Ulcerative colitis (UC) occurs as a result of the interaction among intestinal microbiota, the intestinal barrier, and the immune system. The current treatment options have their limitations and come with side effects, thus there is an urgent need to develop new drug candidates for the treatment of UC. Modified Jiaoqi powder (MJQP) is a Traditional Chinese Medicine formulation improved from an empirical prescription prescribed by Tietao Deng, a celebrated master of Traditional Chinese Medicine. Although MJQP is a viable alternative medication for treating UC, the underlying mechanism is unclear yet.

**Methods:**

Eight groups were designed: control, model, mesalazine group (400 mg/kg), MJQP low-, medium-, and high-dose (2.5, 5, 10 g/kg), AhR antagonist CH223191 (10 mg/kg) combined administration with MJQP (5 g/kg), and CH223191 group (10 mg/kg). The efficacy of MJQP was evaluated using the disease activity index (DAI), colonic length, and pathological alterations. RT-PCR, western blotting, alcian blue staining, immunofluorescence and transmission electron microscopy were used to detect tight junction proteins. The proportion and function of ILC2 and ILC3 were detected by flow cytometry and ELISA, and AhR signaling was examined by western blotting and RT-PCR. 16S rDNA sequencing and targeted tryptophan metabolite detection were used to detect the intestinal microflora and tryptophan metabolites.

**Results:**

MJQP substantially restored body weight and colonic length in UC, and reduced DAI and colonic pathological alterations, while MJQP restored tight junctions of the colon to repair the intestinal barrier. Moreover, MJQP increased the levels of IL-22 and the proportion of IL-22^+^ ILC3, while decreasing the proportion of IL-13^+^ ILC2 and the generation of IL-13. Furthermore, MJQP up-regulated AhR and CYP1A1 expression and down-regulated the expression of ST2. However, these effects were hardly observed when MJQP was administered in combination with an AhR antagonist. Most strikingly, MJQP restored the abundance of *Lachnospiraceae, Lactobacillus* and *Clostridium*, and increased the gut microbiota derived tryptophan metabolites Indole-3-acetic acid (IAA) and Indole-3-propionic acid (IPA), which serve as endogenous AhR ligands to enhance AhR signaling.

**Conclusion:**

MJQP repaired the damaged intestinal barrier to facilitate the resolution of chronic colitis by modulating AhR-mediated ILC2/ILC3 balance through gut microbiota-related tryptophan metabolism.

## Graphical abstract

**Figure:**
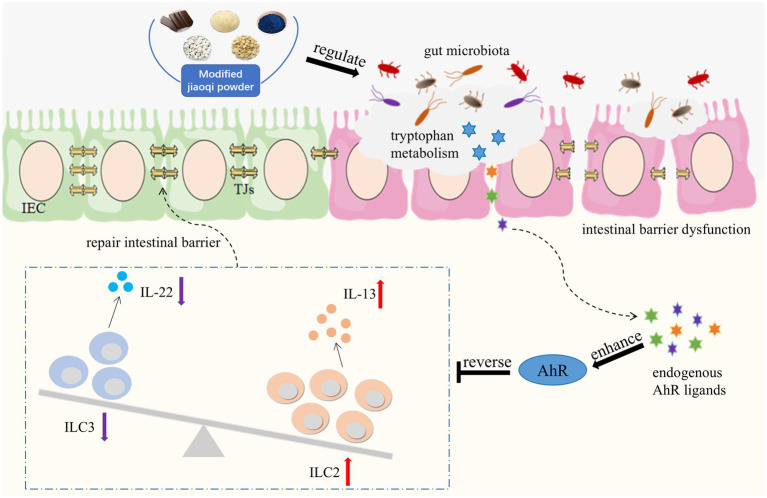

## 1 Introduction

As a nonspecific chronic inflammatory colorectal disease, ulcerative colitis (UC) is characterized by recurrent inflammation and a protracted disease course, along with various complications and a high risk of canceration in severe cases (Wijnands et al., 2021). Some scientists anticipate that there will be 5 million instances of ulcerative colitis worldwide in the future year, and the global incidence and prevalence is still steadily growing (Le Berre et al., 2023). The prevailing view suggests that UC is strongly implicated in intricate pathogenesis, such as dysregulated immune response, intestinal microbiota dysbiosis, genetic susceptibility, and intestinal barrier defects (Gros and Kaplan, 2023). The existing remedies for UC include aminosalicylates, glucocorticoids, immunomodulators and biologics, with the aim of suppressing immune and inflammatory responses, which are not satisfactory because of their adverse effects and tolerance issue resulting from long-term use (Li et al., 2025). Recently, the dysregulation of the interaction between the gut microbiota, the intestinal immune system, and the intestinal barrier play a crucial role in the pathogenesis and progression of UC, making it a highly promising therapeutic target for UC (Han et al., 2025; Li et al., 2025).

A group of immune cells that accumulate in the gastrointestinal tract, called innate lymphoid cells (ILCs) serve as coordinators between adaptive and innate immunity to maintain intestinal immune homeostasis and intestinal barrier repair. ILCs can be classified into ILC1, ILC2, and ILC3 according to the transcription factors and cytokines that they express (Luo et al., 2022; Yao et al., 2025). Under steady conditions, the dominant ILC3 secrete a large amount of IL-22, which is a crucial cytokine in mucosal repair by promoting intestinal epithelial cell (IECs) renewal, stimulating proliferation of Paneth cells and goblet cells to produce antimicrobial peptides and mucins to form a mucosal layer, facilitating the production of epithelial tight junction proteins (TJs) (Yu et al., 2018; Zhou and Sonnenberg, 2020). However, during active inflammation, the amount of ILC3 and their IL-22 secretion decreased significantly, whereas ILC2 increased and secreted larger amounts of pro-inflammatory factors, IL-13 and IL-5 (Forkel et al., 2019), which leads to overexpression of Claudin2 protein and triggers the apoptosis of IECs, further disrupting the epithelial TJs and ultimately exacerbating intestinal barrier damage of ulcerative colitis (Giuffrida et al., 2019; Lin et al., 2019). When the ILCs subset profile changes, intestinal inflammation occurs, which disrupts intestinal homeostasis. The research suggested that in the blood samples and intestinal mucosa of UC patients, the proportion of ILC2 increased while the proportion of ILC3 decreased (Mitsialis et al., 2020; Wu and Shen, 2020).

Prior research has demonstrated that aryl hydrocarbon receptor (AhR) signaling regulates the function of ILC2 and ILC3 to maintain their dynamic balance (Li et al., 2018). AhR is a ligand-dependent transcription factor that regulates the transcription of several target genes, such as CYP1A1 and IL-22, after activation by ligands from dietary and microbial sources (such as tryptophan metabolites) (Kang et al., 2025). Intestinal microbiota dysbiosis and impaired tryptophan metabolism have been confirmed in various autoimmune disease, such as ulcerative colitis, rheumatoid arthritis, systemic lupus erythematosus (Huang et al., 2025; Jian et al., 2025; Radhakrishnan et al., 2025), and a reduction of AhR endogenous ligands was found in the feces in colitis patients (Forkel et al., 2019). Previous studies have proved that that the abundance of gut microbiota related to tryptophan metabolism is reduced, and tryptophan metabolism is impaired, which reduces IL-22 secretion and leading to severe intestinal barrier damage in UC mice (Wang X. et al., 2023; Zhang et al., 2024; Kang et al., 2025; Zuo et al., 2025). Therefore, the gut microbiota-tryptophan metabolism-AhR signaling modulating-ILC2/ILC3 axis may exert a central role in regulating the intestinal mucosal immune homeostasis. However, the immune regulatory mechanism of this axis in intestinal barrier damage of UC has not been reported yet.

Modified Jiaoqi powder (MJQP) composed of *Colla Corii Asini* Pingstone, *Panax notoginseng* Burkill, *Astragalus mongholicus* Bunge, *Wolfiporia cocos* Ryvarden & Gilb and *Strobilanthes cusia* Kuntze, is a traditional Chinese medicine formulation originated from an empirical prescription created by Tietao Deng, a celebrated master of Traditional Chinese Medicine in China. Clinical experience shows that MJQP has a curative effect on promoting ulcer healing and reducing clinical symptoms and recurrence rates in patients with chronic UC. Clinical investigations have demonstrated that the original formulation promotes mucosal healing and inhibits gastrointestinal bleeding (Zhang et al., 2015), and its potential to alleviate acute UC via the regulation of Th17/Treg cell balance and intestinal barrier repair has been illustrated (Wen et al., 2021). Besides, our previous study revealed that MJQP restores the intestinal epithelial barrier by enhancing epithelial autophagy against TNF-mediated apoptosis to ameliorate chronic UC (Liu et al., 2025). However, there is no literature available regarding whether MJQP can treat chronic UC by regulating intestinal mucosal immunity or restoring the intestinal microbiota. This study aimed to investigate therapeutic effects and underlying mechanisms of MJQP in chronic colitis mice, with special attention on their role in regulating the immune balance of the intestinal tract and the composition of the intestinal microbiota.

## 2 Materials and methods

### 2.1 Chemicals and reagents

*Astragalus mongholicus* Bunge and *Wolfiporia cocos* Ryvarden & Gilb were bought from Tiancheng prepared herbal medicine Co., Ltd. (Guangdong, China; batch number 221201 and 220901); *Colla Corii Asini* Pingstone was purchased from Shandong Dong-E E-Jiao Co., Ltd. (Shandong, China; batch number 2208003); *Panax notoginseng* Burkill was obtained from Baiyunshan Pharmaceutical Co., Ltd. (Guangzhou, China; batch number YPB2K0001); *Strobilanthes cusia* Kuntze was supplied from Qianfang Chinese Herb Slices Co., Ltd. (Sichuan, China; batch number 202204026). Mesalazine was procured from Shanghai Aidifa Pharmaceutical Co. Ltd. (Shanghai, China). Dextran sodium sulfate (DSS) was purchased from MP Biomedicals (San Diego, United States). CH223191 was obtained from MedChemExpress (New Jersey, United States). The GoldHi Plasmid Mini Kit was procured from Cowin Biotech Co. Ltd. (Jiangsu, China). Goat anti-mouse/rabbit antibodies were obtained from Bioss Biotechnology Co. Ltd. (Beijing, China). Mouse LPS, IL-22 and IL-13 ELISA kits were supplied by Enzyme Industry Co. Ltd. (Jiangsu, China). AhR, Occludin, ST2, Claudin2, ZO-1 and CYP1A1 antibodies were purchased from Proteintech (Chicago, IL, USA). CD16/32 and fluorescein isothiocyanate (FITC)-lineage antibodies were procured from Tonbo Biosciences (San Diego, United States). PE-CD45, Brilliant Violet 421-GATA3, APC-IL-13, and PerCP/Cy5.5-IL-22 antibodies were purchased from Biolegend (San Diego, United States). The PE-Cy7-RORγt antibody was purchased from Thermo Fisher Scientific (Waltham, MA, USA). All antibodies used were anti-mouse.

### 2.2 Preparation of MJQP

MJQP is composed of *Astragalus mongholicus* (12 g), Wolfiporia cocos (12 g), Colla Corii Asini (3 g), Panax notoginseng (6 g), and Strobilanthes cusia (2 g). MJQP was prepared as follows: *Astragalus mongholicus*, Wolfiporia cocos and Strobilanthes cusia were submerged in distilled water for 30 min and decocted twice for 40 min. Then, Panax notoginseng and Colla Corii Asini were added to dissolve completely, and the liquid was concentrated to produce MJQP, which had a 1 g/mL crude drug concentration.

### 2.3 UPLC–MS/MS

1 mL of the sample was added to 2 mL of the extraction solution of methanol-acetonitrile (1:1, v/v) and ultrasonicated for 30 min. The supernatant was collected after centrifugation (12,000 rpm, 4 °C, 20 min). The sample was incubated at −20 °C for 1 h, then centrifuged (12,000 g, 4 °C, 10 min). The supernatant was dried in a vacuum centrifuge and then re-dissolved in 100 μL of 30% ACN for UPLC–MS/MS analysis.

UPLC-Orbitrap-MS system (UPLC, Vanquish, MS, and HFX) was used to analyses the sample extracts. The UPLC conditions were as follows: column, Waters HSS T3 (100*2.1 mm, 1.8 μm); injection volume, 2 μL; flow rate, 0.3 mL/min; column temperature, 40 °C; solvent system, water (0.1% Formic acid): acetonitrile (0.1% Formic acid); gradient program, 100: 0 V/V at 0–1 min, 5: 95 V/V at 12 min, 5: 95 V/V at 12–13 min, 100: 0 V/V at 13.1–17 min. A Q Exactive HFX Hybrid Quadrupole Orbitrap mass spectrometer system (Thermo Fisher Scientific) was used to LC-MS/MS analysis.

### 2.4 Animals

A total of 80 male C57BL/6 mice (approximately eight weeks age) were supplied by the Guangdong Medical Laboratory Animal Center (Guangdong, China). The experimental animals were nourished on a conventional diet and water and kept in specific pathogen-free surroundings at a temperature of 22 °C ± 2 °C, humidity of 55% ± 2% and a 12-h light/dark cycle. Animal studies were conducted in strict compliance with the Ethics Committee of Animal Experiments of The First Affiliated Hospital of Guangzhou University of Chinese Medicine. Ethical clearance number: GZTCMF1-2023004.

### 2.5 Construction of chronic colitis model

The following groups were designed: control, model, positive drug group (mesalazine at 400 mg/(kg i.g. d), MJQP low-, medium-, and high-dose [2.5, 5, 10 g/(kg i.g. d)] groups, AhR antagonist CH223191 (10 mg/kg i.p. d) combined administration with MJQP (5 g/(kg i.g. d) (CH + M) group, and AhR antagonist CH223191 group (10 mg/kg i.p. d). All mice were randomly assigned to these groups. Chronic UC mice were induced by freely drinking water containing 2% DSS for 4 days following by water for 2 days, for 4 cycles in total, referring to the modeling procedure reported by Huang (Huang et al., 2022). All the mice were anesthetized by intraperitoneal injection of 0.3% pentobarbital sodium and then euthanized by cervical dislocation.

### 2.6 Pharmacodynamic evaluation

Throughout the experimental period, weight loss, diarrhea, and fecal blood of all mice in different groups were recorded on alternate days to monitor and evaluate the changes in the disease activity index (DAI), as previously described (Cooper et al., 1993). On the last day, the spleen and thymus of the mice were separated after euthanisation. Colonic length was measured and organ indices were computed.

### 2.7 Pathological evaluation of colon

The colon fraction was preserved for 24 h by submerging in 4% paraformaldehyde. Subsequently, the samples were prepared in accordance with standard procedures for hematoxylin and eosin staining (H&E) and then observed using an inverted microscope (Olympus IX73).

### 2.8 RT-PCR

The colon was ground in TRIzol reagent using a cryogenic tissue grinder (SCIENTZ-48 L). The GoldHi Plasmid Mini Kit was used to extract the total RNA. Next, the RNA was mixed with 4× RT Master Mix and gDNA Remover reagent for reverse transcription to cDNA. Finally, 2× Color SYBR and primers (shown in Table 1) were added to cDNA, and the mixture was amplified by real-time qPCR (Applied Biosystems QuantStudio 5).

**Table 1:** Sequence of primers for RT-PCR.

Gene	GeneBank accession numbers	Forward primer sequence (5′–3′)	Reverse primer sequence (5′–3′)	qPCR product length (bp)
ZO-1	NM_003257	GCCGCTAAGAGCACAGCAA	TCCCCACTCTGAAAATGAGGA	134
Occludin	NM_001438048	GTCCACCTCCTTACAGACCT	CTGGCTGAGAGAGCATCGG	105
Claudin2	NM_001410421	AAGCAAACAGGCTCCGAAGA	ATGTGCCTAACAGCCCCAAA	106
Muc2	NM_023566	GAAGCCAGATCCCGAAACCA	GAATCGGTAGACATCGCCGT	81
AhR	NM_013464	GTCGCTGCCCTTCATGTTTG	TTGGTGCGTATTGGTAGGGG	96
CYP1A1	NM_001136059	CGTCAGCATCCTCTTGCTACT	TGGCTACTGACACGACCAAAT	132
ST2	NM_001420351	CCGCCTAGTGAACACACCAT	GTCCTCTTTGGGGGCATCTC	211
Il-22	NM_016971	GTTGACACTTGTGCGATCTCT	GCGGTTGACGATGTATGGCT	187
Il-13	NM_008355	GGCAGCATGGTATGGAGTGT	TTTTGGTATCGGGGAGGCTG	169
GAPDH	NM_001411843	GGAGAGTGTTTCCTCGTCCC	GATGGGCTTCCCGTTGATGA	245

### 2.9 Western blotting

For extraction of colonic protein, colon tissue was treated with moderate RIPA containing 1% PMSF, homogenized in a cryogenic tissue grinder (SCIENTZ-48 L), centrifuged, and then the supernatant was collected, which contained the colonic protein. After routine electrophoresis and transfer, the membranes were sealed with 5% skim milk and incubated with the corresponding primary antibodies, such as AhR antibody (1:2000 dilution), CYP1A1 antibody (1:2000 dilution), ST2 antibody (1:2000 dilution), ZO-1 antibody (1:5000 dilution), Occludin antibody (1:5000 dilution) and Claudin2 antibody (1:2000 dilution), and secondary antibodies (1:10000 dilution). Finally, the membrane images were exposed using a chemiluminescence imager (Bio-Rad, Chemidoc MP) and analyzed using the ImageJ software.

### 2.10 Alcian blue staining

After dewaxing the paraffin sections of colon, 30 μL alcian blue solution was added to the sections for staining; the sections were washed with distilled water three times, each time for 10 s; 30 μL nuclear fast red solution was added for staining, then washed with tap water for 5 min. After the section underwent routine dehydration process, it was covered with 20 μL of neutral balsam for sealing, and then observed under an inverted optical microscope (Olympus IX73).

### 2.11 Immunofluorescence

The paraffin sections of colon were routinely dewaxed, immersed in 10 mM sodium citrate buffer and heated for antigen retrieval. After 0.3% Triton was added for permeabilization and 10% goat serum was added to seal, the tissues were incubated with primary antibodies. The next day, the tissues were examined using a fluorescence microscope (Olympus IX73) after being treated with the matching fluorescent secondary antibodies and coated with DAPI.

### 2.12 Flow cytometry

Cell suspensions of mesenteric lymph nodes were prepared by mechanical grinding and filtered through 100, 70 μm cell filtration membranes for collection a flow tube. The CD16/32 antibody blocks nonspecific binding of Fc receptors before cell labeling. The surface markers of ILCs include lneage^−^ (which includes CD11b, CD3, Ly-6G, and B220) and CD45^+^, which were stained using FITC-lineage antibody and PE-CD45 antibody. Cells were then fixed and permeabilized, and nuclear staining was performed with antibodies, including Brilliant Violet 421-GATA3, APC-IL-13, PerCP/Cy5.5-IL-22, and PE-Cy7-RORγt. Finally, the proportions of IL-13^+^ ILC2 and IL-22^+^ ILC3 were analyzed by flow cytometry (FACS LSRFortessa).

### 2.13 Cytokines determination

The secretion levels of TNF-*α*, IL-1β, LPS, IL-13 and IL-22 were determined using ELISA kits.

### 2.14 16S rDNA sequencing and targeted tryptophan metabolites detection

16S rDNA sequencing and targeted tryptophan metabolite detection in fecal samples were conducted by the Shanghai BIOTREE Biomedical Technology Co., LTD (Shanghai, China). Briefly, the 16S rRNA gene V4 was targeted for DNA extraction from fecal samples, and an amplified library was generated for bilateral sequencing using Illumina MiSeq technology. Trimmomatic was used to filter the sequenced raw reads. Next, to obtain high-quality reads, primer sequences were located and eliminated. The overlapping sequences were subsequently spliced using FLASH v1.2.7 software to acquire clean reads. To obtain the final valid data for further analysis (splitting features, diversity analysis, species analysis, difference analysis, and correlation analysis), chimera sequences were examined and eliminated using UCHIME software.

For metabolite extraction, fecal samples were weighed and approximately 50 mg was placed into a tube. The samples were homogenized (35 Hz, 4 min) and sonicated for 5 min after 500 μL of the extract solution was added. After repeating the homogenate and sonication cycle twice, the temperature was lowered to −40 °C. Following 15-min centrifugation (12,000 rpm, 4 °C), 320 μL of the supernatant was transferred to another tube. Subsequently, the supernatant was dried off under a nitrogen stream and then mixed with 80 μL of 0.1% formic acid for reconstitution. After a 15-min centrifugation (12,000 rpm, 4 °C), UHPLC–MS/MS analysis was conducted on the supernatants.

### 2.15 Statistical analysis

All data were analyzed with GraphPad Prism 8.3 software and shown as mean ± standard deviation (SD). Two separate groups were compared using Student’s *t*-test, and group comparisons were conducted using one-way analysis of variance (ANOVA). The threshold for a statistically significant difference was *p* < 0.05.

## 3 Results

### 3.1 Chemical component analysis of MJQP

**Figure 1:**
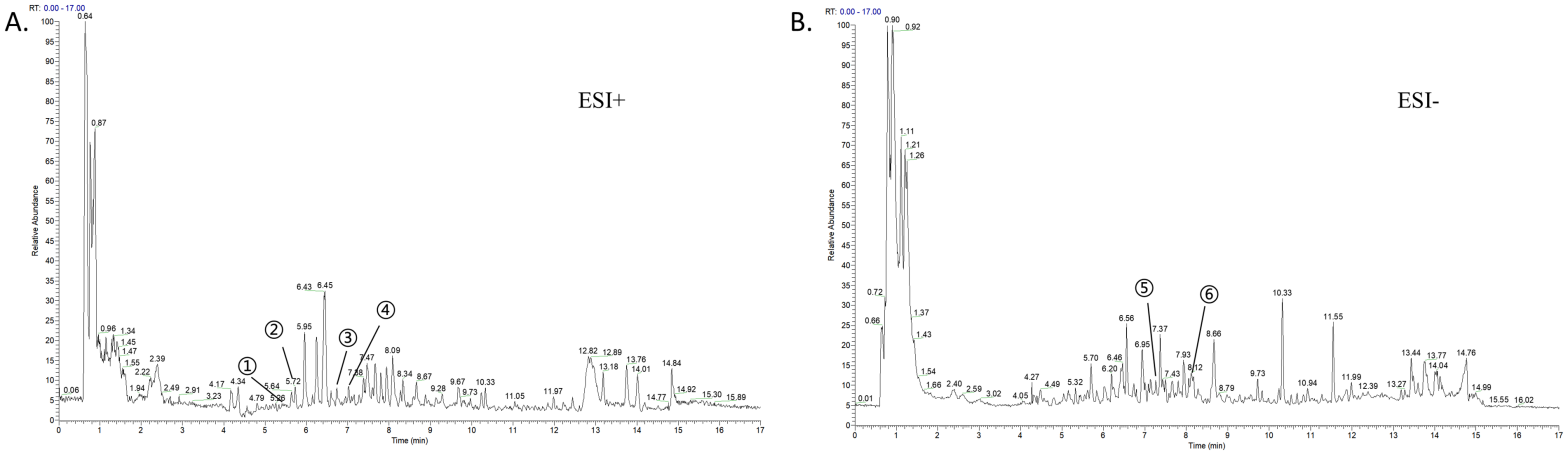
Chemical component analysis of MJQP. **(A)** Positive extracting ion chromatogram; **(B)** Negative extracting ion chromatogram. 1. Quercetin; 2. Calycosin-7-*O*-beta-D-glucoside; 3. Ononin; 4. Formononetin; 5. Liquiritigenin; 6. Kaempferol.

To characterize the material basis of MJQP, UPLC-MS/MS analysis was used. Figures 1A,B showed the positive and negative extracting ion chromatogram of MJQP. The compounds were characterized by matching with retention time, molecular mass, and secondary fragmentation spectra in databases. The results showed that prenol lipids, fatty acyls, organooxygen compounds, flavonoids, carboxylic acids and derivatives were main components with percentage as 25.7%, 11.19%, 8.92%, 7.17%, and 6.12%, respectively. Among them, the main chemical components were flavonoids, such as quercetin, ononin, calycosin-7-O-*β*-D-glucoside, formononetin, liquiritigenin, kaempferol. The full lists of flavonoids in MJQP were shown in Supplementary Table S1.

### 3.2 MJQP ameliorated the symptoms of chronic colitis mice

**Figure 2:**
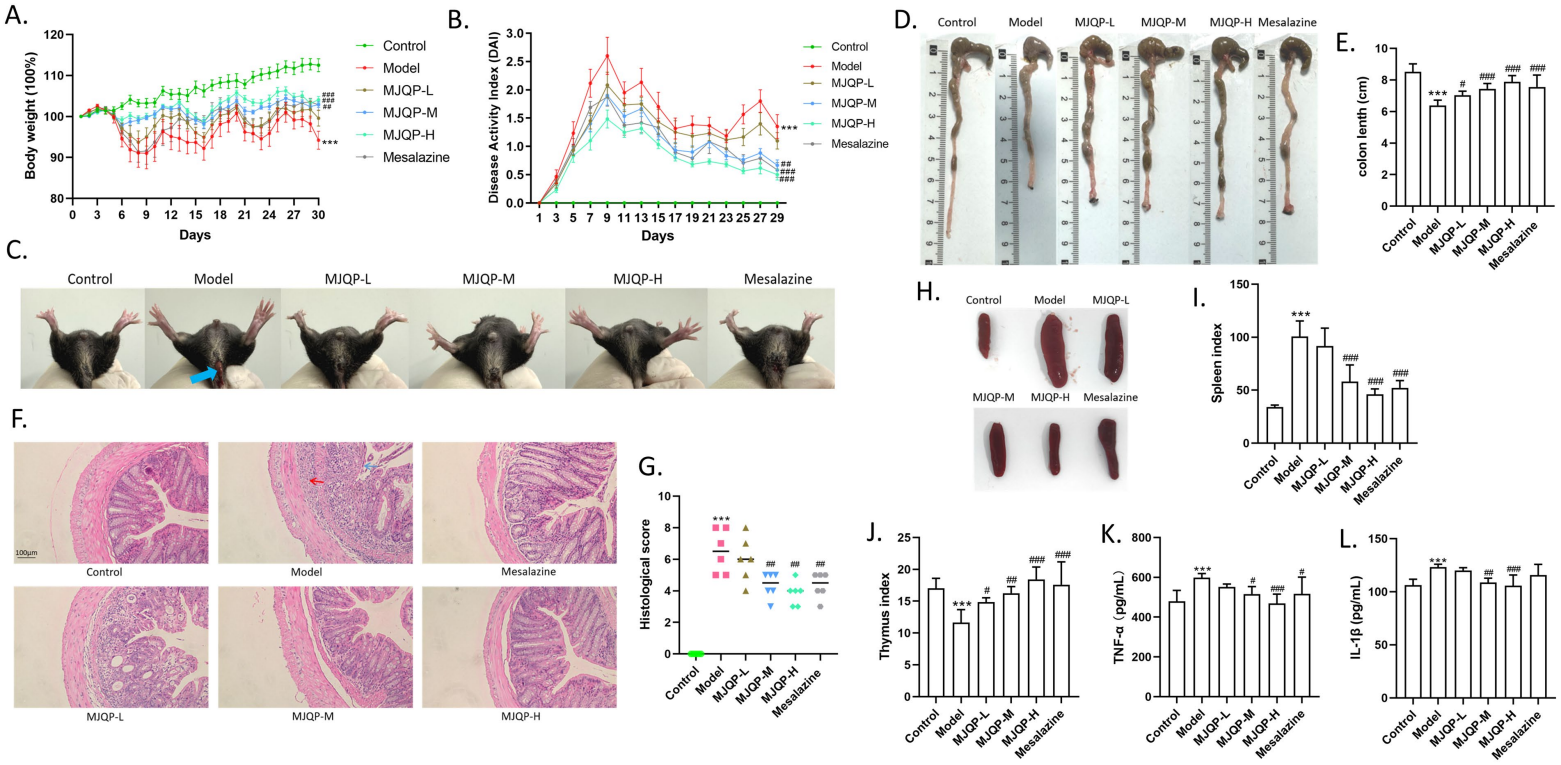
MJQP ameliorated the symptoms of chronic colitis mice. **(A)** Body weight; **(B)** DAI scores; **(C)** Stool bleeding; **(D,E)** Length of colons; **(F,G)** Colonic histopathological score (200×); **(H,I)** Spleen index; **(J)** Thymus index; **(K,L)** The levels of TNF-*α* and IL-1β in the serum. *n* = 5–8. ***p < 0.001 vs. the control group. ^#^*p* < 0.05, ^##^*p* < 0.01, ^###^*p* < 0.001 vs. the model group.

To assess the effect of MJQP in chronic colitis mice, we treated them with three different doses of MJQP and mesalazine. Compared with mice in the control group, DSS resulted in classic UC symptoms in model mice, such as bloody stool, significant weight-decrease, increased DAI score, decreased thymus index, increased spleen index, constricted colon (a marker of inflammation), and the elevated levels of pro-inflammatory cytokines TNF-*α* and IL-1β in the serum. By sharp contrast, MJQP and mesalazine administration improved the weight loss, constricted colon and thymus index, reduced DAI score and spleen index, and decreased the levels of TNF-α and IL-1β in the serum (Figures 2A–E,H–L). In addition, UC-related colonic histological alterations were found in model mice, including erosion and ulceration, epithelial cell depletion, and the muscularis with deep mucosal lymphocytosis (Figures 2F,G), which were relieved in medium and high-dose of MJQP as well as mesalazine-treated mice, along with a lower histological score as compared to the model mice (Figures 2F,G).

### 3.3 MJQP improved the damaged intestinal barrier in chronic colitis mice

**Figure 3:**
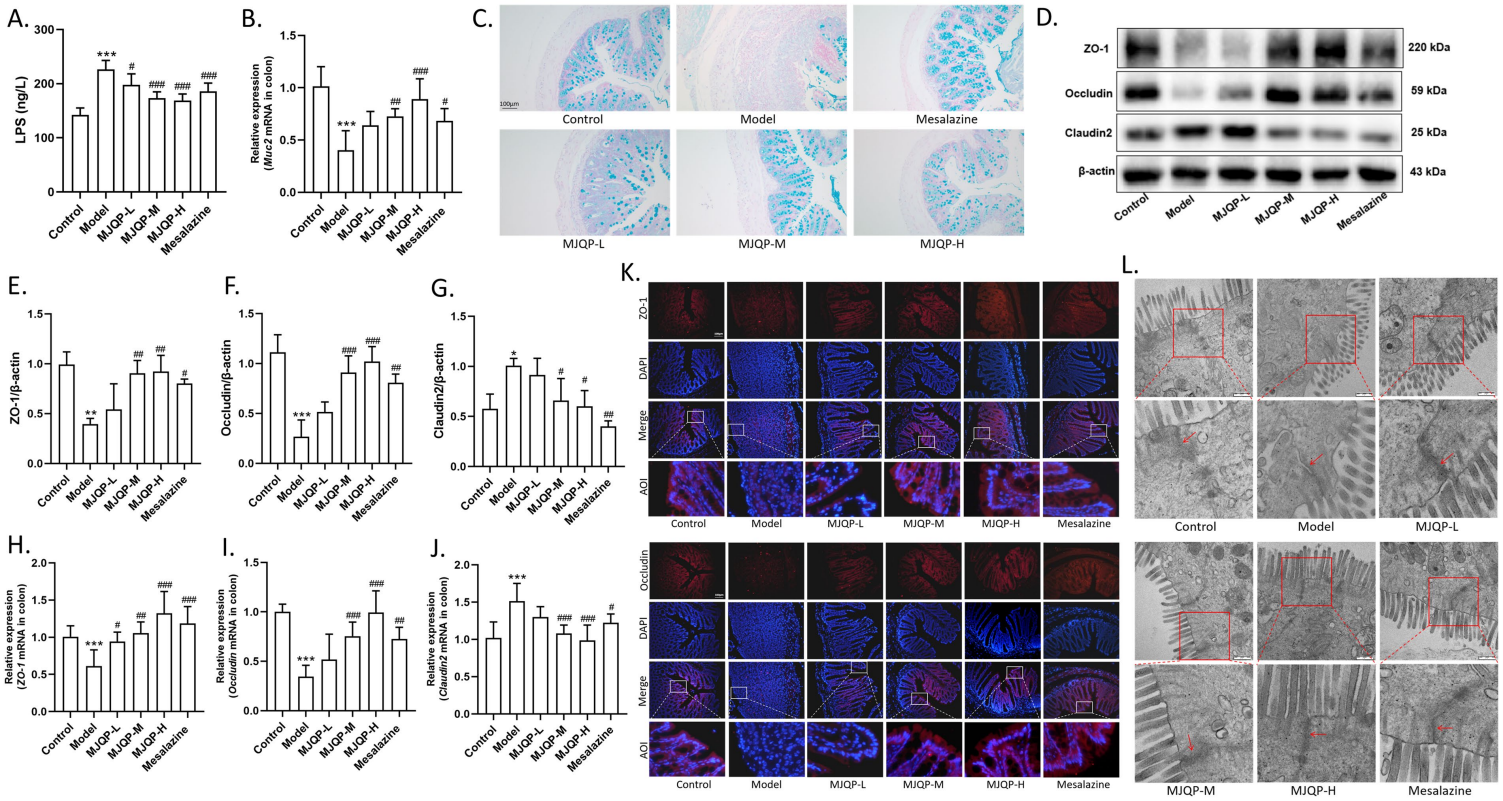
MJQP improved the damaged intestinal barrier in chronic colitis mice. **(A)** LPS content in serum; **(B)** *Muc2* mRNA expression; **(C)** Mucins (blue) determined by Alcian blue staining (200×). **(D–G)** The expression of TJ proteins ZO-1, occludin, and claudin2; **(H–J)** The mRNA levels of *ZO-1*, *occludin, and claudin2*; **(K)** Immunofluorescence detection for ZO-1 (red) and occludin (red) in colon (200×); **(L)** Tightness of intestinal epithelium tight junctions were observed by transmission electron microscopy (40000×), the red box represents the magnified area. *n* = 3–6. *p < 0.05, **p < 0.01, ***p < 0.001 vs. the control group. ^#^*p* < 0.05, ^#^*p* < 0.01, ^###^*p* < 0.001 vs. the model group.

According to the ELISA results, model mice had higher levels of LPS in response to DSS stimulation, which decreased after treatment with MJQP and mesalazine (Figure 3A). Moreover, mice in model group showed significantly reduced Muc2 mRNA expression and mucin secretion (Figures 3B,C), as well as the epithelial TJ rupture presented as decreased levels of ZO-1, Occludin mRNA and protein, and increased level of Claudin2 (Figures 3D–K). The intestinal epithelial integrity was effectively enhanced by MJQP and mesalazine compared with the model group, which promoted Muc2 mRNA expression and mucin secretion (Figures 3B,C), down-regulated the mRNA and protein expression of Claudin2, and up-regulated the expression of ZO-1 and Occludin (Figures 3D–K). Transmission electron microscopy (Figure 3L) consistently showed TJ repair in the intestinal epithelium of MJQP-treated mice, indicating that MJQP is capable of restoring the intestinal barrier integrity. Together, the aforementioned findings showed that MJQP repaired intestinal barrier damage to alleviate chronic colitis in mice.

### 3.4 MJQP regulated the ILC2/ILC3 balance and AhR signaling in chronic colitis mice

**Figure 4:**
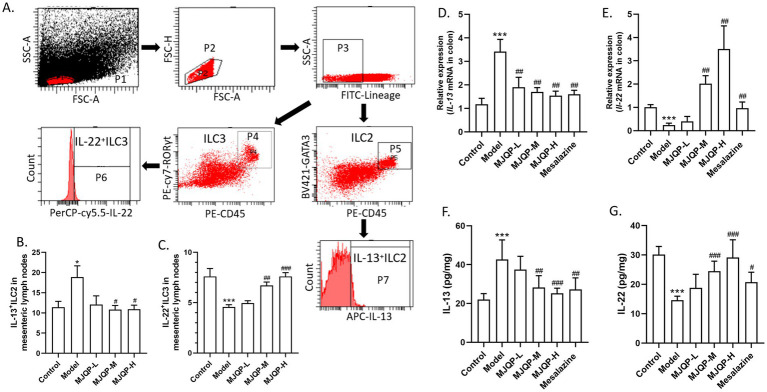
MJQP regulated the ILC2/ILC3 balance in chronic colitis mice. **(A)** Logical plot of the flow cytometry gate; **(B,C)** the IL-13^+^ ILC2 and IL-22^+^ ILC3 percentage detected by flow cytometry; **(D,E)** the mRNA levels of *Il-13* and *Il-22* in colon were determined by RT-PCR; **(F,G)** the content of IL-13 and IL-22 in colon tissue homogenate were tested by ELISA. *n* = 3–6. *p < 0.05, ***p < 0.001 vs. the control group. ^#^*p* < 0.05, ^##^*p* < 0.01, ^###^*p* < 0.001 vs. the model group.

Innate lymphocytes are crucial participants in mediating pathogen immunity and maintaining intestinal immune homeostasis (Saez et al., 2021). ILC2/ILC3 imbalance leads to aberrant release of the cytokines IL-13 and IL-22, which causes intestinal disruption. Therefore, we examined how ILC2, ILC3 and their effectors change in the colons of chronic colitis mice. The proportion of IL-22^+^ ILC3 decreased dramatically, whereas that of IL-13^+^ ILC2 increased noticeably according to the flow cytometry results (Figures 4A–C). Correspondingly, the levels of IL-22 significantly reduced while IL-13 contents raised in colon (Figures 4D–G). Following medium and high-dose of MJQP intervention, there was a discernible increased ratio of IL-22^+^ ILC3, as well as a corresponding decreased ratio of IL-13^+^ ILC2 in chronic colitis mice (Figures 4A–C). Moreover, MJQP dose-dependently decreased IL-13 contents, and increased IL-22 contents (Figures 4D–G).

**Figure 5:**
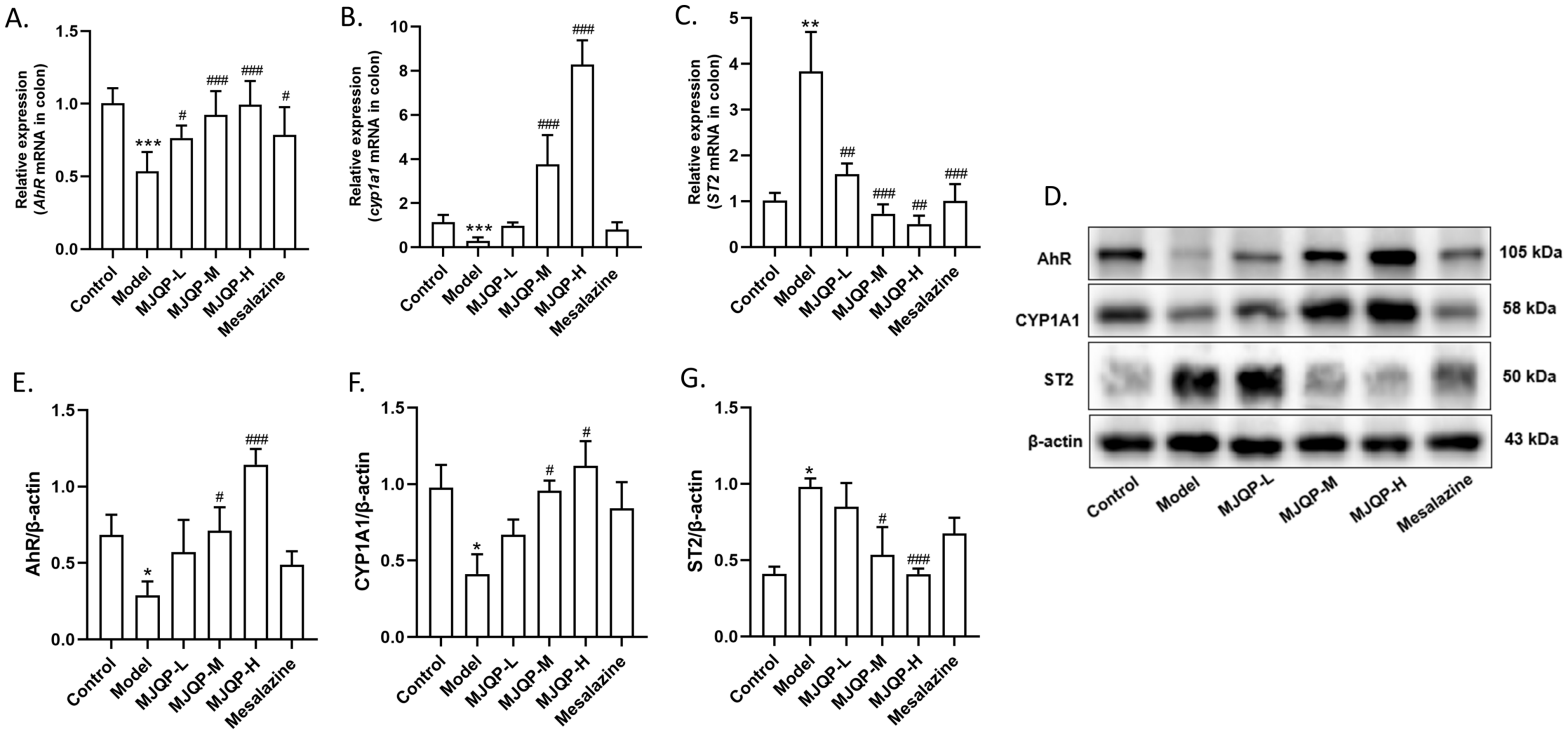
MJQP modulated AhR signaling pathway in chronic colitis mice. **(A–C)** The mRNA levels of *AhR*, *CYP1A1,* and *ST2*; **(D–G)** the protein levels of AhR, CYP1A1, and ST2. *n* = 3–6. *p < 0.05, **p < 0.01, ***p < 0.001 vs. the control group. ^#^*p* < 0.05, ^##^*p* < 0.01, ^###^*p* < 0.001 vs. the model group.

A previous study reported that AhR controls the function of ILC2 and ILC3 to maintain their balance (Li et al., 2018). Therefore, we investigated the effects of MJQP on AhR signaling in mice with chronic colitis. Compared to the control group, the expression of AhR and CYP1A1 (AhR’s downstream target gene) in the colon of the model group were significantly decreased, whereas ST2 expression was increased. MJQP could reverse these changes in a dose-dependent fashion (Figures 5D–G). Correspondingly, MJQP-treated groups displayed decreased *ST2* mRNA expression and elevated *AhR* and *CYP1A1* mRNA expression in comparison to the model group (Figures 5A–C). These findings indicated that MJQP regulates the AhR signaling-mediated ILC2/ILC3 balance, which be related to its beneficial effects on the intestinal barrier.

### 3.5 AhR antagonists weakened the effect of MJQP on intestinal barrier repair

**Figure 6:**
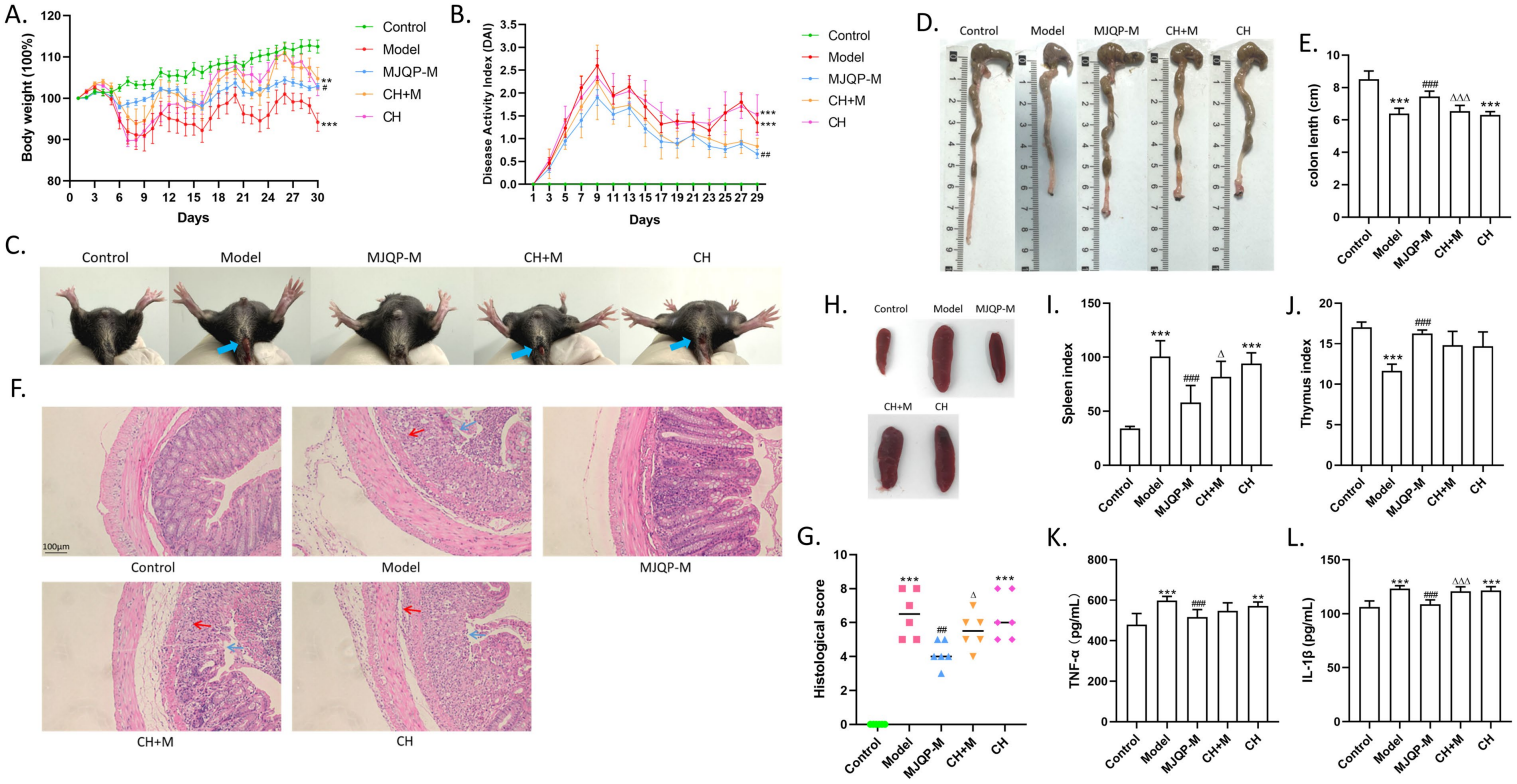
AhR antagonists weakened MJQP’s effect on improving symptoms in chronic colitis mice. **(A)** Body weight; **(B)** DAI scores; **(C)** Stool bleeding; **(D,E)** Length of colons; **(F,G)** Colonic histopathological score (200×); **(H,I)** Spleen index; **(J)** Thymus index; **(K,L)** The levels of TNF-α and IL-1β in the serum. *n* = 5–8. **p < 0.01, ***p < 0.001 vs. the control group. ^#^*p* < 0.05, ^##^*p* < 0.01, ^###^*p* < 0.001 vs. the model group. ^△^*p* < 0.05, ^△△△^*p* < 0.001 vs. the MJQP-M group.

**Figure 7:**
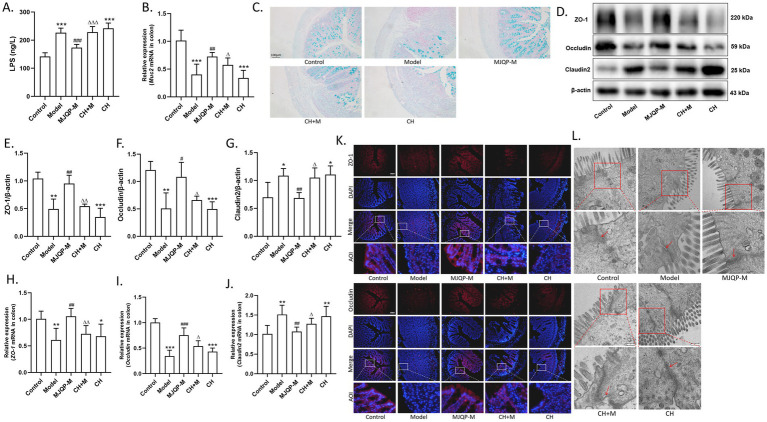
AhR antagonists weakened the effect of MJQP on intestinal barrier repair. **(A)** LPS content in serum; **(B)** *Muc2* mRNA expression; **(C)** Mucins (blue) determined by Alcian blue staining (200×). **(D–G)** The expression of TJ proteins ZO-1, occludin, and claudin2; **(H–J)** The mRNA levels of *ZO-1*, *occludin, and claudin2*; **(K)** Immunofluorescence detection for ZO-1 (red) and occludin (red) in colon (200×); **(L)** Tightness of intestinal epithelium tight junctions were observed by transmission electron microscopy (40000×), the red box represents the magnified area. *n* = 4–6. *p < 0.05, **p < 0.01, ***p < 0.001 vs. the control group. ^#^*p* < 0.05, ^##^*p* < 0.01, ^###^*p* < 0.001 vs. the model group. ^△^*p* < 0.05, ^△△^*p* < 0.01, ^△△△^*p* < 0.001 vs. the MJQP-M group.

We then added the AhR antagonist CH223191 as a pretreatment before MJQP administration (CH + M) to investigate whether the effect of MJQP on intestinal barrier repair is AhR-dependent. The result showed that mice in the CH + M group showed more severe UC symptoms compared to the MJQP-treat group. In particular, AhR antagonists resulted in increased DAI score, constricted colons, considerable spleen swelling, and the elevated levels of pro-inflammatory cytokines TNF-*α* and IL-1β in the serum (Figures 6A–E,H–L). Meanwhile, H&E staining result showed that CH223191 weakened MJQP’s effect on eliminating ulcerative erosion, reducing mucosal lymphocyte infiltration and improving mucosal structure (Figures 6F,G). In addition to the observations above, CH223191 also increased the contents of LPS in serum of mice (Figure 7A). Besides, disrupted intestinal barrier structures were observed in CH + M group mice, which were correlated with considerably decreased mucin expression (Figures 7B,C), lower levels of ZO-1, Occludin expression, and higher levels of Claudin2 expression in comparison to the MJQP administration group (Figures 7D–K). Consistent with the protein results, transmission electron microscopy revealed the TJ of intestinal epithelium to be breaking down (Figure 7L). These results implied that AhR is necessary for MJQP to repair intestinal barrier damage in chronic colitis mice.

### 3.6 AhR antagonists reversed the effects of MJQP on regulation of ILC2/ILC3 balance and AhR signaling enhancement

**Figure 8:**
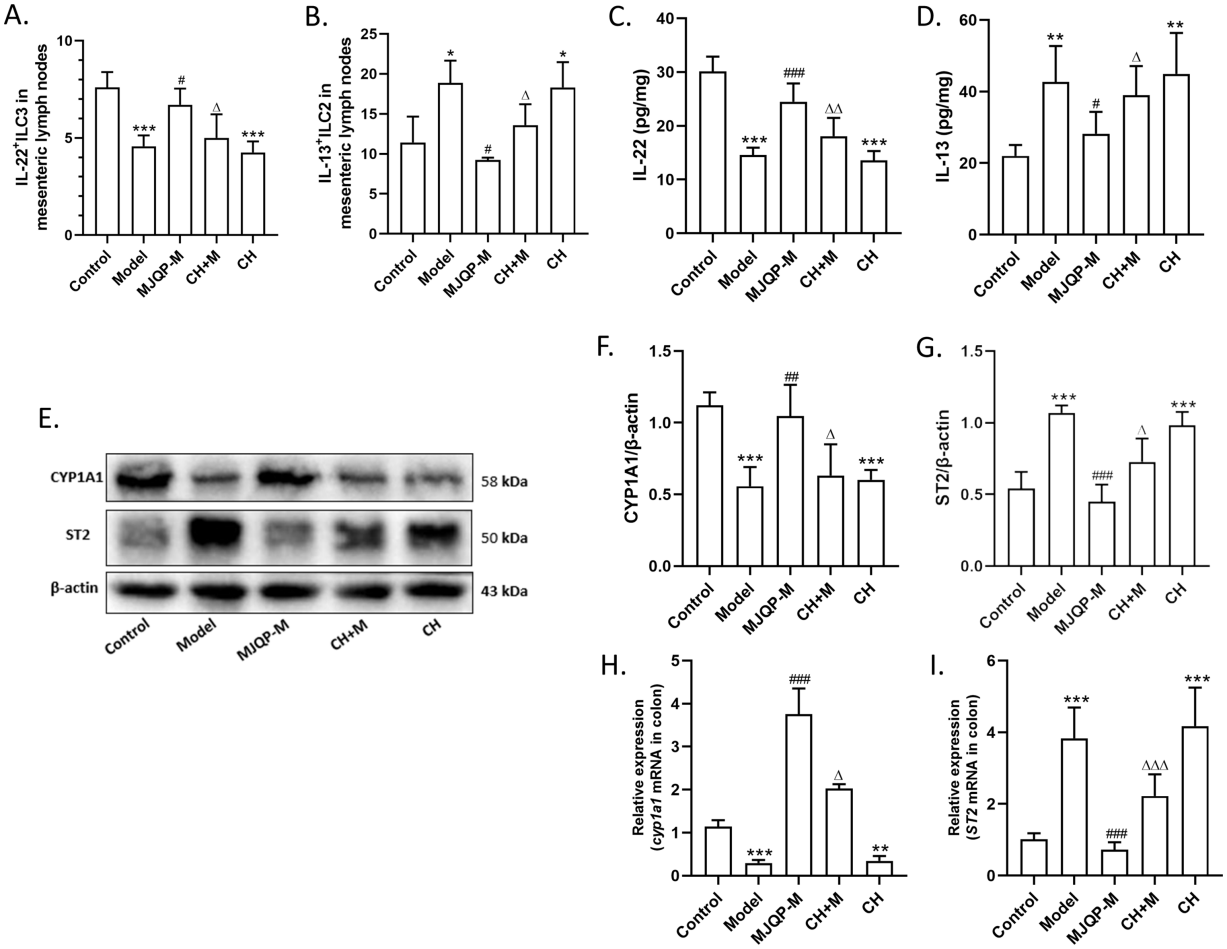
AhR antagonists reversed the effects of MJQP on regulation of ILC2/ILC3 balance and AhR signaling enhancement. **(A,B)** The IL-22^+^ ILC3 and IL-13^+^ ILC2 percentage detected by flow cytometry; **(C,D)** The content of IL-22 and IL-13 in colon; **(E–G)** The protein levels of CYP1A1 and ST2, *n* = 4; **(H,I)** The mRNA levels of *CYP1A1* and *ST2*, *n* = 5–6. ^*^p < 0.05, **p < 0.01, ***p < 0.001 vs. the control group. ^#^*p* < 0.05, #^##^*p* < 0.01, ^###^*p* < 0.001 vs. the model group. ^△^*p* < 0.05, ^△△^*p > p* < 0.01, ^△△△^*p* < 0.001 vs. the MJQP-M group.

We further examined whether AhR was required for MJQP-modulated ILC2/ILC3 balance. As shown in Figures 8A–D, AhR antagonist CH223191 combined administration with MJQP (CH + M) could significantly elevate IL-13^+^ ILC2 proportion and IL-13 contents, while reduced IL-22^+^ ILC3 proportion and IL-22 levels when compared to MJQP-treated group. Moreover, CH223191 significantly increased the protein and mRNA expression of ST2, while obviously decreased the expression of CYP1A1 in the colon of the CH + M group compared with the MJQP-treated group (Figures 8E–I). The results collectively suggested that the role of MJQP in modulating ILC2/ILC3 balance to repair intestinal barrier in chronic colitis mice is dependent on AhR signaling pathway.

### 3.7 MJQP restored intestinal microbiota-driven tryptophan metabolites in chronic colitis mice

**Figure 9:**
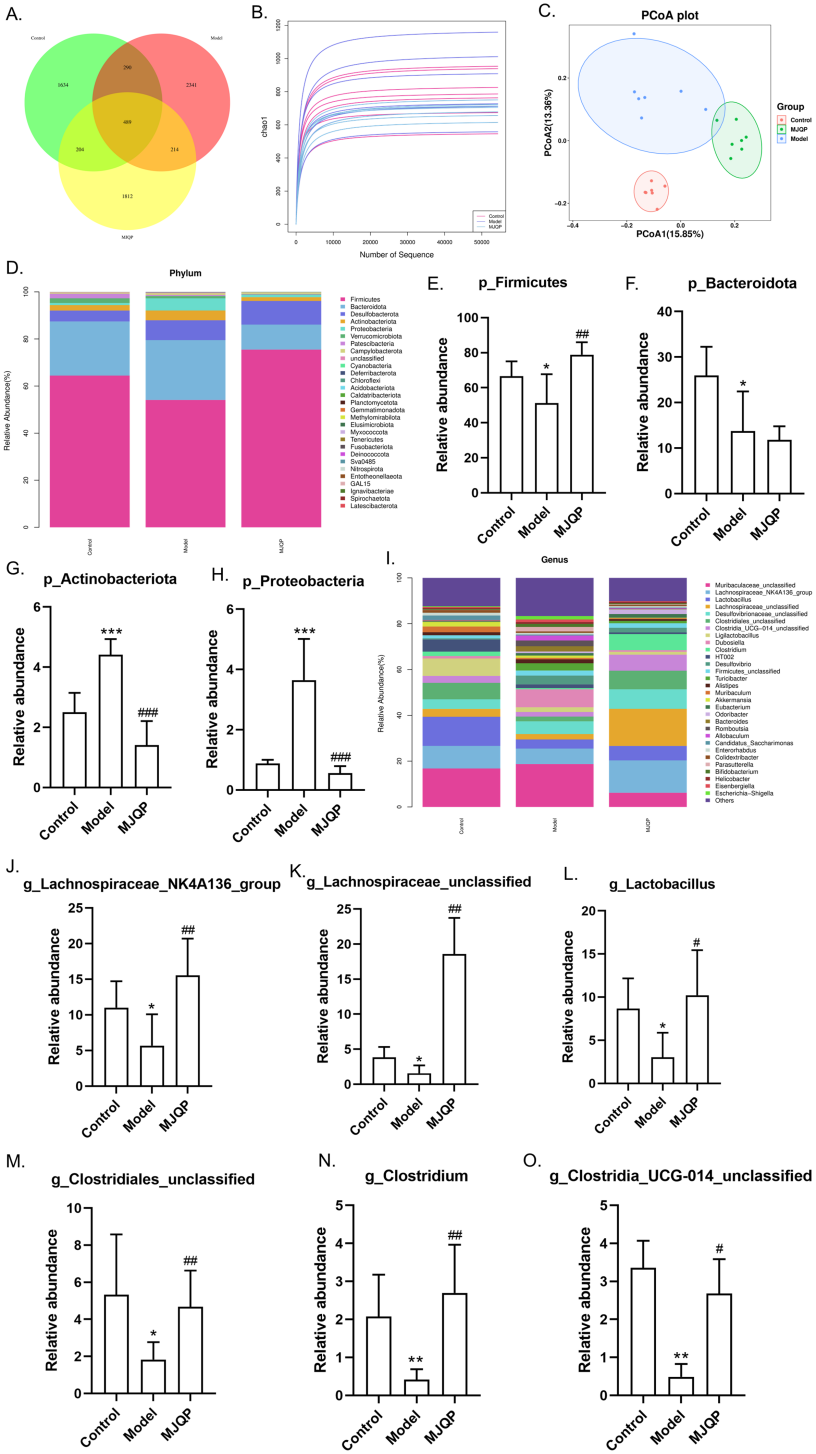
MJQP altered the composition of intestinal microbiota. **(A)** Venn diagram of gut microbiota; **(B)** Chao1 curve diagram; **(C)** PCoA plots assessed by UnwUF; **(D)** Relative abundance histograms at phylum; **(E–H)** Difference analysis of gut microbiota at phylum levels; **(I)** Relative abundance histograms at genus; **(J–O)** Difference analysis of gut microbiota at genus levels. *n* = 4–6. *p < 0.05, **p < 0.01, ***p < 0.001 vs. the control group. ^#^*p* < 0.05, ^##^*p* < 0.01, ^###^*p* < 0.001 vs. the model group.

The primary endogenous ligand sources of AhR are indole derivatives with AhR activity, which originate from tryptophan, metabolized by the intestinal microbiota. To explore whether MJQP enhances the AhR signaling pathway by restoring tryptophan metabolites from the gut microbiota, we examined variations in the intestinal microbiota composition and tryptophan metabolites. As illustrated in the Venn diagram, the control, model, and MJQP groups had 1,634, 2,341, and 1812 individual operational taxonomic units (OTUs) respectively, and shared 489 identical OTUs (Figure 9A). The rarefaction curve directly reflects the sufficiency and richness of a sample. As shown in Figure 9B, the curve tended to be flat, indicating that the sample size was reasonable and species-rich. Besides, principal coordinate analysis (PCoA) showed that the intestinal microbiota structure varied considerably between the control and colitis groups, while there was little difference between the two colitis groups (Figure 9C). We further examined the bacterial composition. As depicted in Figures 9D–H, compared to the control group at the phylum level, the reduced abundance of *Firmicutes* and *Bacteroidetes*, and the elevated abundance of *Actinobacteria* and *Proteobacteria* were observed in the model group. However, MJQP markedly augmented the abundance of *Firmicutes* and diminished that of *Actinobacteria* and *Proteobacteria* compared to the model group. When compared with the model group, the abundance of *Lachnospiraceae*_*NK4A136*_*group*, *Lactobacillus*, *Lachnospiraceae*_*unclassified*, *Clostridiales*_*unclassified*, *Clostridia*_*UCG-014*_*unclassified*, *Clostridium* were considerably rised in MJQP group at the genus level (Figures 9I–O), and that of *Escherichia-Shigella*, *Coriobacteriaceae*_*unclassified*, *Bifidobacterium*, *Erysipelatoclostridium* and *Bacteroides* were observably reduced (Supplementary Figure S1). Noteworthily, *Lachnospiraceae*, *Lactobacillus*, *Clostridia* and *Clostridiales* are essential for the metabolism of tryptophan.

**Figure 10:**
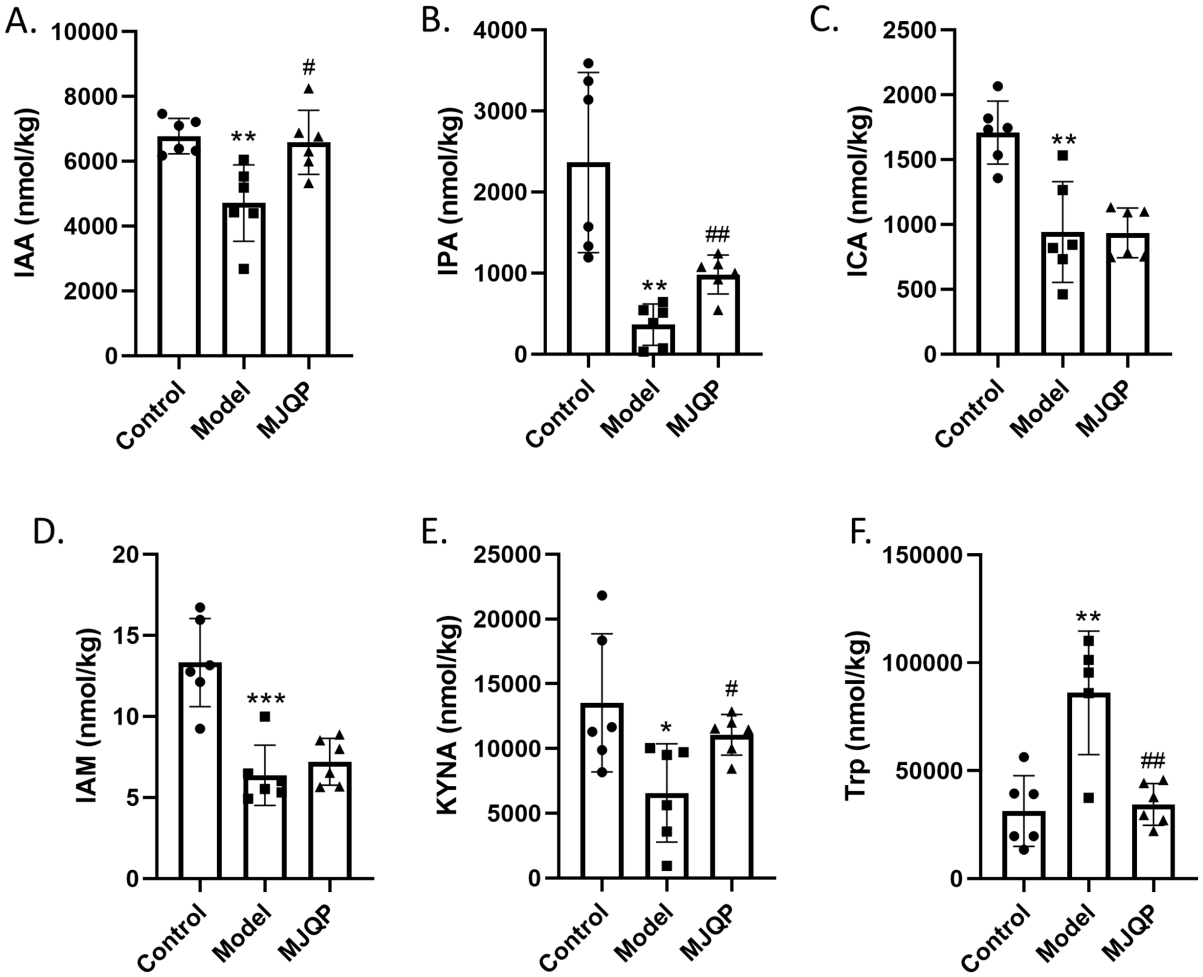
MJQP restored intestinal microbiota-driven tryptophan metabolites. **(A–F)** Quantification of tryptophan and its metabolites in mice feces. IAA, Indole-3-acetic acid; IPA, Indole-3-propionic acid; ICA, Indole-3-carboxaldehyde; IAM, Indole-3-acetamide, KYNA, Kynurenic acid; Trp, L-Tryptophan. *n* = 5–6. *p < 0.05, **p < 0.01, ***p < 0.001 vs. the control group. ^#^*p* < 0.05, ^#^*p* < 0.01 vs. the model group.

**Figure 11:**
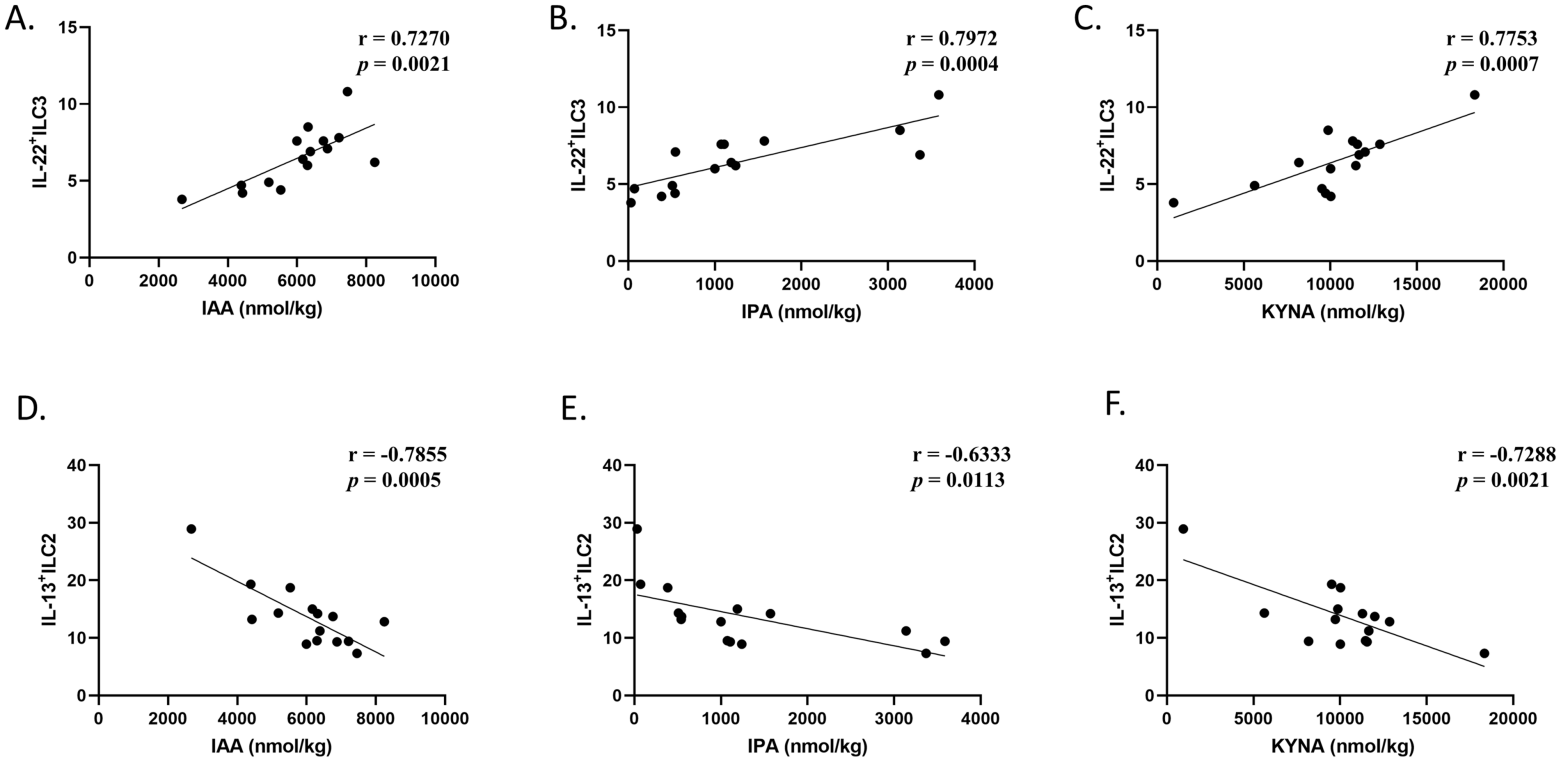
Correlation analysis of tryptophan metabolites with IL-22^+^ ILC3, IL-13^+^ ILC2. **(A–C)** Correlation analysis of IAA, IPA, KYNA and IL-22^+^ ILC3; **(D–F)** correlation analysis of IAA, IPA, KYNA and IL-13^+^ ILC2. *r* > 0 indicates that the two groups of data are positively correlated, *r* < 0 indicates that the two groups of data are negatively correlated. |*r*| ≤ 0.3, no linear correlation; 0.3 ≤ |*r*| ≤ 0.5, low linear relationship; 0.5 ≤ |*r*| ≤ 0.8, significant linear relationship; |*r*| > 0.8, height linear relationship.

We next observed alterations in tryptophan metabolites (particularly indole derivatives) associated with the intestinal microbiota in the mouse feces. In the model group, the level of Indole-3-acetic acid (IAA), Indole-3-propionic acid (IPA), Indole-3-carboxaldehyde (ICA), Indole-3-acetamide (IAM), and kynurenic acid (KYNA) were decreased, and that of L-tryptophan (Trp) was upregulated compared to the control group (Figures 10A–F), implying that DSS-induced chronic colitis mice suffered tryptophan metabolism disturbances related to the gut microbiota. After MJQP intervention, the concentrations of IAA, IPA, and KYNA were increased, and that of Trp was reduced in comparison with the model group, indicating that MJQP may enhance AhR signaling by restoring tryptophan metabolites (as endogenous AhR ligands) associated with the gut microbiota. There were no significant changes in the levels of the other tryptophan metabolites (Supplementary Figure S2). Additionally, the correlation analysis of IAA, IPA, KYNA, IL-13^+^ ILC2, IL-22^+^ ILC3 were executed, respectively. The results indicated that IL-22^+^ ILC3 were positively correlated with IAA, IPA, and KYNA (Figures 11A–C). On the other hand, negative correlations were discovered between IL-13^+^ ILC2 and the aforementioned metabolites (Figures 11D–F). Collectively, MJQP restored intestinal microbiota-driven tryptophan metabolites, which could act as potential endogenous ligands of AhR to enhance the AhR pathway and regulate ILC2/ILC3 balance to achieve therapeutic effects in chronic colitis.

## 4 Discussion

Accumulative researches have revealed that the development and recurrence of ulcerative colitis is associated with structural intestinal barrier breakdown. As a result, mucosal healing has become the main focus of UC treatment and is a crucial sign in the clinical diagnosis and prognostic evaluation of UC (Neurath and Vieth, 2023; Rath et al., 2023). In this study, DSS caused chemical disruption of the intestinal epithelium, inducing a chronic colitis model accompanied by a variety of typical UC symptoms, such as weight loss, diarrhea, and bloody stool; constricted colon caused by colonic myoedema, eroded colon mucosa and the inflamed lamina propria infiltrated by immune cells; spleen swelling and thymic atrophy triggered by immune disorders (Nascimento et al., 2020). Therefore, the model was utilized to evaluate the efficacy of MJQP, and the results showed that MJQP substantially relieved the above symptoms, which confirmed the therapeutic effect of MJQP in mice with chronic colitis.

We then explored whether MJQP affects the intestinal barrier based on pharmacodynamics. The normal intestinal epithelium acts as a barrier that prevents potentially harmful microorganisms from invading tissues, moderates inflammatory reactions, and facilitates the absorption of nutrients. Intestinal permeability increases, and luminal microorganisms infiltrate the intestinal epithelium as the intestinal barrier is breached (Li et al., 2022; Wang X. et al., 2023). Meanwhile, bacterial endotoxins, which are inflammatory mediators (such as LPS), migrate via the bloodstream to other tissues or organs and contribute to extraintestinal inflammatory illness (Forlano et al., 2022). Consequently, we confirmed that MJQP could improve intestinal barrier function in chronic colitis mice as reflected in lower levels of serum LPS. We investigated MJQP’s effect on the intestinal epithelial structure, since intestinal barrier function is linked to the integrity of the epithelial structure, which is influenced by tight junction proteins (TJs). Patients with colitis have poor intestinal barrier function when the TJs are disrupted. Likewise, UC mice exhibit reduced expression of ZO-1 and Occludin (Schlegel et al., 2021; Wang X. et al., 2023). Furthermore, intestinal epithelial mucin formation prevents intestinal bacteria from directly interacting with IECs and invading the intestinal lamina propria to avoid excessive inflammatory responses. In our study, MJQP improved the damaged intestinal barrier as manifested by the upregulation of TJs and increased mucin expression.

ILCs are essential participants in intestinal repair because of their strategic accumulation in the intestinal lamina propria and their prompt and effective response to intestinal epithelial damage and invading pathogens (Thomas and Peebles, 2022; Ryu et al., 2023). Among them, ILC3 are crucial for maintaining intestinal homeostasis by responding quickly to tissue damage, inflammation, and infection (Li et al., 2023). During homeostasis or tissue injury, ILC3 release the signature pro-repair cytokine IL-22, which binds to heterodimeric receptors on the surface of IECs, IL-22R1 and IL-10R2, activating the STAT3 and NOD2 signaling pathways to produce mucins and antimicrobial peptides, and promotes the expression of TJ proteins to preserve intestinal integrity (Schulz-Kuhnt et al., 2021). Prior research has claimed that in a *C. rodentium*-infected colitis model mice receiving anti-IL-22 antibodies or IL-22 knockout mice had lower survival rates (Lee et al., 2011). The number of ILC3 in the colon tissue was considerably reduced, which correlated with the severe UC of colon endoscopy were (Mitsialis et al., 2020). However, a significant amount of ILC2 was detected in the colon tissue of UC patients, and its effector IL-13 also increased (Forkel et al., 2019). ILC2 aggravates intestinal injury by secreting IL-13 disrupting epithelial TJs, triggering epithelial cell apoptosis, and increasing the expression of Claudin2 (Heller et al., 2005). We found that MJQP decreased the ratio of IL-13^+^ ILC2, while upregulating that of IL-22^+^ ILC3, suggesting that it regulates the ILC2/ILC3 balance to create an immune microenvironment favorable for intestinal barrier repair.

Previous research has confirmed that AhR maintains the intestinal barrier function by regulating homeostasis of the intestinal immune microenvironment, and it can serve as an important regulator of ILC2/ILC3 balance (Li et al., 2018). It is worth noting that AhR signaling is necessary for ILC3 maintenance and development. Specifically, AhR recruits IL-22 promoters under the cooperation of RORγt, thereby regulating IL-22 gene transcription and secretion, and AhR deletion leads to the obstruction of this process. Likewise, AhR-knockout mice are more susceptible to developing colitis because of their insufficient ILC3 and inadequate IL-22 production (Qiu et al., 2012). Conversely, AhR inhibits ILC2 function by preventing ST2 expression (IL-33 receptor), which hinders the release of its effector molecule, IL-13. Increased IL-33 levels and ST2 expression (UC biomarkers) are consistent with the pro-inflammatory characteristics of type 2 immunity (such as IL-13) in UC (Liu et al., 2022). Moreover, ILC3 percentage at the inflammatory site of the colon was lower than that at the non-inflammatory site in patients with UC, accompanied by decreased expression and activity of AhR (Forkel et al., 2019). MJQP inhibited the expression of ST2 and elevated AhR and CYP1A1 expression (AhR’s downstream target gene), revealing that MJQP enhanced the AhR signaling pathway. Moreover, in order to investigate whether AhR is required for MJQP to control the function of ILC2/ILC3 accounting for beneficial effect to alleviate UC, AhR antagonist CH223191 was used to block the AhR signaling pathway. As expected, CH223191 impeded the repairing effect of MJQP on the intestinal barrier and hindered restoration of the ILC2/ILC3 balance mediated by MJQP. Our study indicated that MJQP combats chronic colitis mice by enhancing AhR signaling to modulate ILC2/ILC3 balance.

In addition, we explored how MJQP enhances AhR signaling. As a ligand-dependent transcription factor, enhancement of AhR signaling is mainly attributed to AhR ligand activation. AhR ligands come from three different sources: plant-based ligands, such flavonoids, ligands produced by the metabolism of gut microbiota, and ligands created by host metabolism (Lamas et al., 2018). Among them, most endogenous AhR ligands are indole derivatives, which are derived from tryptophan degraded by intestinal flora and act as the medium mediating host-microbiome crosstalk to cooperatively maintain intestinal homoeostasis. In this study, we found that the fecal tryptophan content of the model group was notably increased, reflecting impaired tryptophan metabolism in colitis mice. However, MJQP increased the abundance of *Lachnospiraceae*, *Lactobacillus* and *Clostridium*, in which *Lactobacillus* and *Clostridium* are known to metabolize tryptophan to produce indole-3-lactic acid (ILA), IAA, and IPA (Wang et al., 2024). Specifically, *Lactobacillus* metabolizes tryptophan into ILA, thereby augmenting the expression of key bacterial enzymes implicated in tryptophan metabolism, leading to the synthesis of IAA and IPA. *Lactobacillus*, as a well-known probiotic in the intestinal tract, has a significantly reduced abundance in the intestines of UC patients. The IAA produced by *Lactobacillus* is not only a ligand for AhR, but also can activate the IL-22/STAT3 signaling pathway in intestinal epithelial cells, in synergy with the AhR signal, to promote the expression of mucin and tight junction proteins (Shi et al., 2020; Zuo et al., 2025). Moreover, numerous studies have shown that restoring the abundance of Lactobacillus can facilitate the conversion of tryptophan into IAA and IPA, thereby activating the AhR signal, reducing colonic inflammation and repairing the intestinal barrier (Yang et al., 2021; Yang et al., 2022; Yang et al., 2023; Jing et al., 2025). *Lachnospiraceae* is also involved in the production of IAA and IPA (Koay et al., 2019; Gao et al., 2023; Wang J. et al., 2023). As a key member of the intestinal commensal microbiota, multiple studies have confirmed that a decrease in its abundance is positively correlated with the severity of intestinal inflammation in UC. This reduction leads to insufficient biosynthesis of IAA and IPA, resulting in hypoactivation of AhR signaling pathway. Conversely, enhancing the abundance of *Lachnospiraceae* and restoring tryptophan metabolism can effectively alleviate UC (Wan et al., 2022; Lv et al., 2024; Xu et al., 2025). Consistent with this, MJQP increased the concentrations of IAA and IPA as endogenous AhR ligands capable of AhR activation in colitis mice feces, which could explain the role of MJQP in enhancing AhR signaling. In addition, MJQP also activated the host-mediated tryptophan metabolism reflected in increased KYNA content, which has been reported to protect against intestinal damage by activating AhR (Wang et al., 2022; Grishanova and Perepechaeva, 2024). Importantly, correlation analysis showed that there was a positive association between the concentrations of IAA, IPA, KYNA and the proportion of IL-22^+^ ILC3, and a negative correlation with IL-13^+^ ILC2. Collectively, our study indicates that MJQP alleviates chronic colitis by enhancing the AhR signaling pathway to regulate ILC2/ILC3 balance through restoration of gut microbiota-related tryptophan metabolites as potential endogenous AhR ligands.

However, AhR is also activated by polycyclic aromatic hydrocarbons such as flavonoids, which are abundant in several plants. The majority of flavonoids serve as exogenous AhR ligands, and research has demonstrated that different flavonoids have different effects on AhR activity based on their structure (Jin et al., 2018). MJQP is a traditional Chinese medicine formulation with complex chemical components, and whether it can activate AhR signaling directly? It is an attractive avenue for further research on the active ingredients of MJQP that target AhR to govern ILC2/ILC3 balance to repair intestinal barrier damage.

## 5 Conclusion

In summary, this study demonstrated that MJQP repairs intestinal barrier damage and exerts a curative effect in chronic colitis mice, and its mechanism is tied to enhancing AhR signaling mediating ILC2/ILC3 functional regulation by restoring intestinal microbiota-driven tryptophan metabolites as endogenous AhR ligands. These findings not only preliminarily evaluated potential of MJQP as a regulator of gut microbiota to generate more endogenous AhR ligands but also shed light on the mechanism by which MJQP alleviates chronic colitis to lay a scientific foundation for the advancement of its clinical application.

## Data Availability

The data presented in the study are deposited in the https://www.ncbi.nlm.nih.gov/, accession number PRJNA1420379.
