# Supplementary material for: Modified Jiaoqi powder ameliorates ulcerative colitis through gut microbiota-tryptophan metabolism-AhR signaling modulating-ILC2/ILC3 balance
Source: Front Microbiol. 2026 Feb 25;17:1764082. doi: 10.3389/fmicb.2026.1764082 (PMC12977975; doi:10.3389/fmicb.2026.1764082)
Supplement: Supplementary file 1 [file Data_Sheet_1.docx]

**Supplementary Table 1 The full lists of flavonoids in MJQP**

| Mode | Components/Metabolite name | Formula | m/z | Retention time (min) |
| --- | --- | --- | --- | --- |
| [H]^-^ | Complanatuside | C_28_H_32_O_16_ | 669.171410 | 4.46 |
| [H]^-^ | Aromadendrin 4'-glucoside | C_21_H_22_O_11_ | 449.109869 | 5.24 |
| [H]^-^ | Kaempferol 3-gentiobioside | C_27_H_30_O_16_ | 609.147812 | 5.47 |
| [H]^-^ | Asebotin | C_22_H_26_O_10_ | 449.146319 | 5.57 |
| [H]^-^ | Hyperoside | C_21_H_20_O_12_ | 463.088934 | 5.77 |
| [H]^-^ | Isoliquiritin | C_21_H_22_O_9_ | 417.120001 | 5.84 |
| [H]^-^ | Apigenin-7-O-Beta-D-glucoside | C_21_H_20_O_10_ | 477.104773 | 5.99 |
| [H]^-^ | Astragalin | C_21_H_20_O_11_ | 447.094280 | 6.03 |
| [H]^-^ | Yuanhuanin | C_22_H_22_O_11_ | 507.115296 | 6.11 |
| [H]^-^ | Phlorizin | C_21_H_24_O_10_ | 435.131041 | 6.33 |
| [H]^-^ | Odoratin-7-O-beta-D-glucopyranoside | C_23_H_24_O_11_ | 521.130209 | 6.59 |
| [H]^-^ | 6,7,4'-Trihydroxyflavanone | C_15_H_12_O_5_ | 271.061401 | 6.64 |
| [H]^-^ | Hydroxygenkwanin | C_16_H_12_O_6_ | 299.056826 | 6.71 |
| [H]^-^ | Alpha-Isowighteone | C_20_H_18_O_5_ | 383.114501 | 6.97 |
| [H]^-^ | Isomucronulatol 7-O-glucoside | C_23_H_28_O_10_ | 463.161661 | 7.09 |
| [H]^-^ | 7,8-Dihydroxyflavone | C_15_H_10_O_4_ | 253.051191 | 7.12 |
| [H]^-^ | Liquiritigenin | C_15_H_12_O_4_ | 255.066673 | 7.26 |
| [H]^-^ | Iristectorigenin B | C_17_H_14_O_7_ | 329.067240 | 7.38 |
| [H]^-^ | Isosakuranetin | C_16_H_14_O_5_ | 285.077344 | 7.47 |
| [H]^-^ | Biochanin A | C_16_H_12_O_5_ | 283.061689 | 7.77 |
| [H]^-^ | Apigenin | C_15_H_10_O_5_ | 269.045762 | 7.97 |
| [H]^-^ | Kaempferol | C_15_H_10_O_6_ | 285.040935 | 8.03 |
| [H]^-^ | Isorhamnetin | C_16_H_12_O_7_ | 315.051715 | 8.15 |
| [H]^-^ | Hispidulin | C_16_H_12_O_6_ | 299.056834 | 8.22 |
| [H]^-^ | Butin | C_15_H_12_O_5_ | 271.061399 | 8.87 |
| [H]^-^ | Acacetin | C_16_H_12_O_5_ | 283.061586 | 15.16 |
| [H]^+^ | Quercetin | C_15_H_10_O_7_ | 303.049157 | 5.44 |
| [H]^+^ | Rhamnocitrin 3-rutinoside | C_28_H_32_O_15_ | 609.180320 | 5.55 |
| [H]^+^ | Calycosin-7-O-Beta-D-glucoside | C_22_H_22_O_10_ | 447.127260 | 5.72 |
| [H]^+^ | Diosmetin-7-O-Beta-D-glucopyranoside | C_22_H_22_O_11_ | 463.122327 | 6.30 |
| [H]^+^ | 6''-O-Acetylglycitin | C_24_H_24_O_11_ | 489.137986 | 6.61 |
| [H]^+^ | Ononin | C_22_H_22_O_9_ | 431.132325 | 6.74 |
| [H]^+^ | Retusine | C_16_H_25_NO_5_ | 329.206119 | 6.85 |
| [H]^+^ | Isomucronulatol 7-O-beta-glucoside | C_23_H_28_O_10_ | 465.174533 | 7.09 |
| [H]^+^ | Formononetin | C_16_H_12_O_4_ | 269.080009 | 7.15 |
| [H]^+^ | Genistein | C_15_H_10_O_5_ | 271.059376 | 7.98 |
| [H]^+^ | Echioidinin | C_16_H_12_O_5_ | 317.101238 | 8.05 |
| [H]^+^ | Licopyranocoumarin | C_21_H_20_O_7_ | 417.153612 | 8.54 |
| [H]^+^ | Haginin A | C_17_H_16_O_5_ | 333.132466 | 8.76 |
| [H]^+^ | Pendulone | C_17_H_16_O_6_ | 299.090686 | 8.80 |


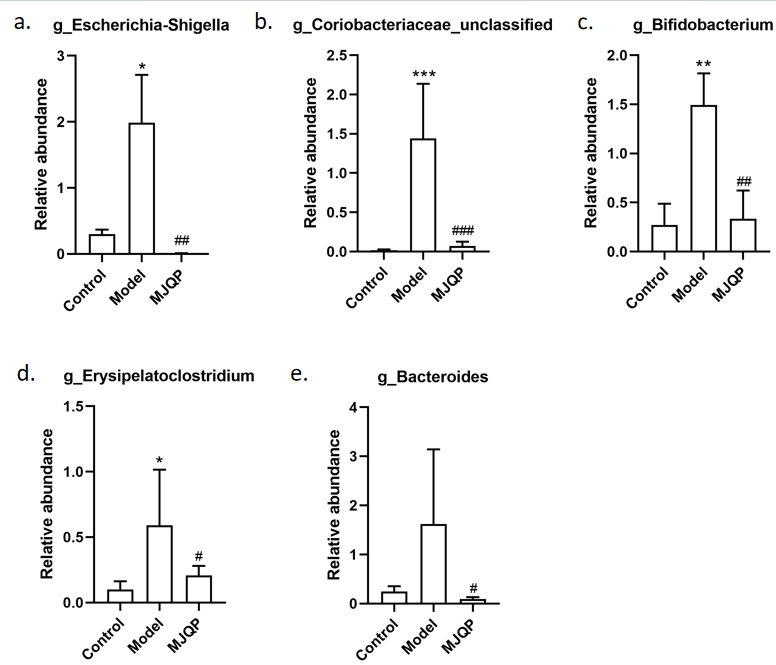
Supplementary Fig. 1

**Supplementary Fig. 1. MJQP reduces the relative abundance of harmful bacteria.** (a-e). Difference analysis of gut microbiota at genus levels (n=5-6). **p* < 0.05, ***p* < 0.01, ****p* < 0.001 vs. the control group. ^#^*p* <0.05, ^##^*p* <0.01, ^###^*p* <0.001 vs. the model group.

Supplementary Fig. 2


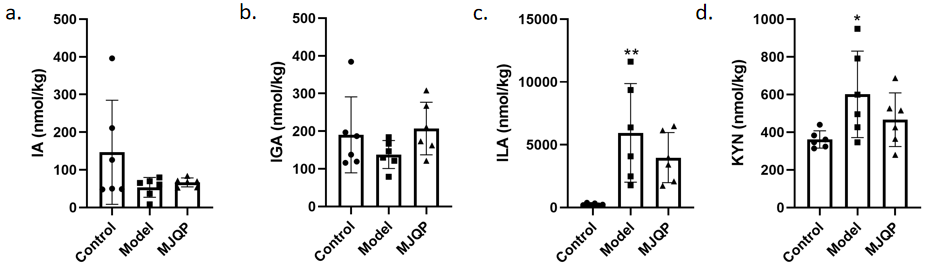
**Supplementary Fig. 2. The other tryptophan metabolites.** (a-d). Quantification of tryptophan and its metabolites in mice feces (n=6). IA, indole acrylic acid; IGA, 3-Indoleglyoxylic acid; ILA, Indolelactic acid; KYN, Kynurenine. **p* < 0.05, ***p* < 0.01 vs. the control group.
